# Four phospholipase A_2_ genes encoded in the western flower thrips genome and their functional differentiation in mediating development and immunity

**DOI:** 10.1038/s41598-024-60522-8

**Published:** 2024-04-29

**Authors:** Mojtaba Esmaeily, Yonggyun Kim

**Affiliations:** Department of Plant Medicals, College of Life Sciences, Andong National University, Andong, 36729 Korea

**Keywords:** Biochemistry, Immunology, Molecular biology

## Abstract

Eicosanoids are synthesized from phospholipids by the catalytic activity of phospholipase A_2_ (PLA_2_). Even though several PLA_2_s are encoded in the genome of different insect species, their physiological functions are not clearly discriminated. This study identified four PLA_2_ genes encoded in the western flower thrips, *Frankliniella occidentalis*. Two PLA_2_s (*Fo-PLA*_2_*C* and *Fo-PLA*_2_*D*) are predicted to be secretory while the other two PLA_2_s (*Fo-PLA*_2_*A* and *Fo-PLA*_2_*B*) are intracellular. All four PLA_2_ genes were expressed in all developmental stages, of which *Fo-PLA*_2_*B* and *Fo-PLA*_2_*C* were highly expressed in larvae while *Fo-PLA*_2_*A* and *Fo-PLA*_2_*D* were highly expressed in adults. Their expressions in different tissues were also detected by fluorescence in situ hybridization. All four PLA_2_s were detected in the larval and adult intestines and the ovary. Feeding double-stranded RNAs specific to the PLA_2_ genes specifically suppressed the target transcript levels. Individual RNA interference (RNAi) treatments led to significant developmental retardation, especially in the treatments specific to *Fo-PLA*_2_*B* and *Fo-PLA*_2_*D*. The RNAi treatments also showed that *Fo-PLA*_2_*B* and *Fo-PLA*_2_*C* expressions were required for the induction of immune-associated genes, while *Fo-PLA*_2_*A* and *Fo-PLA*_2_*D* expressions were required for ovary development. These results suggest that four PLA_2_s are associated with different physiological processes by their unique catalytic activities and expression patterns.

## Introduction

Phospholipase A_2_ (PLA_2_) catalyzes phospholipids (PLs) to release arachidonic acid (AA), which is usually used for the biosynthesis of eicosanoids^1^. Eicosanoids mediate various physiological processes such as immunity, metabolism, and reproduction in metazoans including insects^2^. However, most terrestrial insects possess little AA in their PLs and use linoleic acid, which is subsequently extended and desaturated into AA^3^. In mammals, AA released from PLs is oxygenated into prostaglandins (PGs) by cyclooxygenase (COX)^4^. Insects, which do not have COX orthologs, use a specific peroxidase called peroxinectin for the biosynthesis of PGs^5^. AA is also oxygenated by lipoxygenase (LOX)^6^. LOX or its equivalent enzyme has been not identified in insects. AA is also oxygenated by epoxygenase (EPX) into four different eicosatrienoic acids (EETs) in mammals and insects^7^.

All types of eicosanoids play a crucial role in mediating insect immunity^8^. Upon pathogen infection, the stimulation of sessile hemocytes by PGs induced their mobilization leading to an increase in the total number of circulatory hemocytes within 2 h^9^. In a beetle, *Tribolium castaneum*, RNA interference (RNAi) was systemically applied to suppress specific gene expression with high efficiency^10^. In this system, Toll/IMD signal pathways activated PLA_2_ to produce PGs and LTs, which led to the expression of specific antimicrobial peptides (AMPs) against different pathogens^11^. All four types of EETs were detected in a lepidopteran insect, *Spodoptera exigua*, and were found to mediate both cellular and humoral immune responses^12^. These findings indicate that PLA_2_ catalyzes the committed step for the biosynthesis of eicosanoids and finally mediates the immune responses.

After the discovery of the first PLA_2_ in snake venom, similar disulfide-rich PLA_2_s were also found in mammalian systems^13–16^. The subsequent recognition of non-disulfide bond-containing PLA_2_s from intracellular sources necessitated the classification of PLA_2_s into groups^17^. At least 16 PLA_2_ groups are now recognized, including five major types: secretory PLA_2_s (sPLA_2_s: groups I–III, V, IX, X, XI, XII, XIII, XIV, and XV), calcium-dependent intracellular PLA_2_ (cPLA_2_: group IV), calcium-independent intracellular PLA_2_ (iPLA_2_: group VI), lipoprotein-associated PLA_2_ (LpPLA_2_: groups VII and VIII), and adipose phospholipase A_2_ (AdPLA_2_: group XVI)^18^. sPLA_2_ and LpPLA_2_ are secretory proteins that act on extracellular membrane lipids, while cPLA_2_ and iPLA_2_ catalyze the hydrolysis of fatty acids from intracellular phospholipids. However, the localization of LpPLA_2_ and AdPLA_2_ is not clear.

The western flower thrips, *Frankliniella occidentalis*, is an invasive insect pest that infests various crops^19^. It is also known to transmit a plant virus, tomato spotted wilt virus (TSWV)^20^. The use of chemical insecticides to control this insect pest leads to the development of insecticide resistance^21^. An entomopathogenic fungus, *Beauveria bassiana*, was identified as an effective biological control agent^22^. However, the immune responses of the thrips, which are mediated by eicosanoids, play a crucial role in defending against the virulence of the fungi^23^. Furthermore, *F. occidentalis* also exhibits a potent antiviral response against TSWV involving apoptosis and AMPs via eicosanoid mediation^24^. However, the mechanism of eicosanoid biosynthesis in *F. occidentalis* remains unclear.

This study identified PLA_2_ genes from the *F. occidentalis* genome and analyzed their expressions. Based on their expression profile, the enzyme activities of PLA_2_ were analyzed in different stages of the development of *F. occidentalis*. Individual RNAi treatments were applied to assess their independent physiological roles in the development, immunity, and reproduction of *F. occidentalis*.

## Results

### Variation in PLA_2_ enzyme activities of *F. occidentalis*

**Figure 1:**
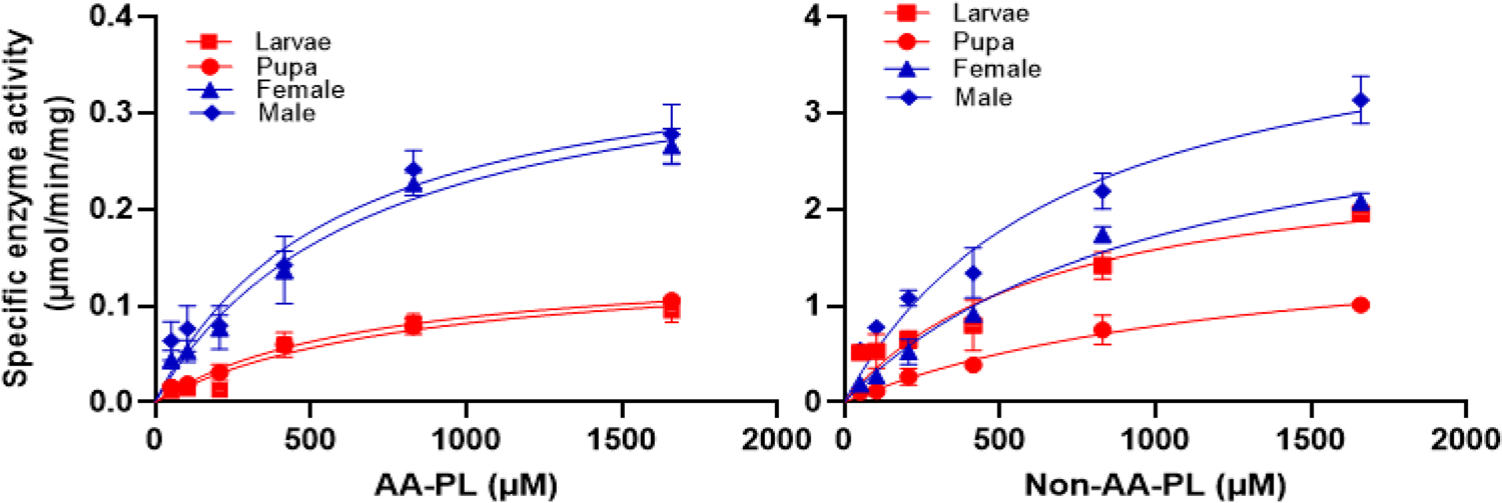
Variation in PLA_2_ activities among the developmental stages of *F. occidentalis*. Change in PLA_2_ activity with increase in substrate amount, in which two different substrates were arachidonate phospholipids (‘AA-PL’) and non-arachidonate phospholipids (‘Non-AA-PL’).

All developmental stages of *F. occidentalis* from larva to adult exhibited PLA_2_ enzyme activities (Fig. 1). PLA_2_ enzymes extracted from different stages catalyzed two different phospholipid substrates (AA-PL and non-AA-PL) in a dose-dependent manner. However, the kinetic parameters of enzyme activities differed between the developmental stages (Table 1). Enzyme affinity to substrate measured by Michaelis–Menten constant (km) varied among different stages (*F* = 3.31; df = 6, 16; *P* = 0.0258) and between two substrate types (*F* = 13.93; df = 1, 16; *P* = 0.0018). Except for the larval stage, other developmental stages preferred AA-PL over non-AA-PL. AA-PL was the most preferred by male adults while non-AA-PL was the most preferred by larvae. The maximal catalytic capacities measured by Vmax varied among different stages (*F* = 9.58; df = 6, 16; *P* = 0.0001) and between two substrate types (*F* = 130.03; df = 1, 16; *P* < 0.0001). PLA_2_s of all developmental stages exhibited higher Vmax values in non-AA-PL (2.0–4.9 μmol/min/μg) than in AA-PL (0.13–0.38 μmol/min/μg). In both substrates, adult PLA_2_s showed higher Vmax values than immature stages.

**Table 1:** Variation in Michaelis–Menten parameters of PLA_2_ enzyme activities in different developmental stages of *F. occidentalis.*

Substrate	Stage	N	km (µM)	Vmax (µmol/min/mg)
AA-PL	Larva	3	1044 ± 14.4^a^	0.17 ± 0.01^b^
	Pupa	3	572 ± 11.5^b^	0.13 ± 0.03^b^
	Male	3	504 ± 12.7^c^	0.38 ± 0.04^a^
	Female	3	506 ± 9.6^c^	0.34 ± 0.03^a^
Non-AA-PL	Larva	3	783 ± 15.5^c^	2.7 ± 0.04^c^
	Pupa	3	1713 ± 17.9^a^	2.0 ± 0.02^d^
	Male	3	1006 ± 23.4^b^	4.9 ± 0.02^a^
	Female	3	1036 ± 14.5^b^	3.40 ± 0.02^b^

Different letters following standard deviations indicate significant differences in mean values for each substrate at Type I error = 0.05 (LSD test).

**Figure 2:**
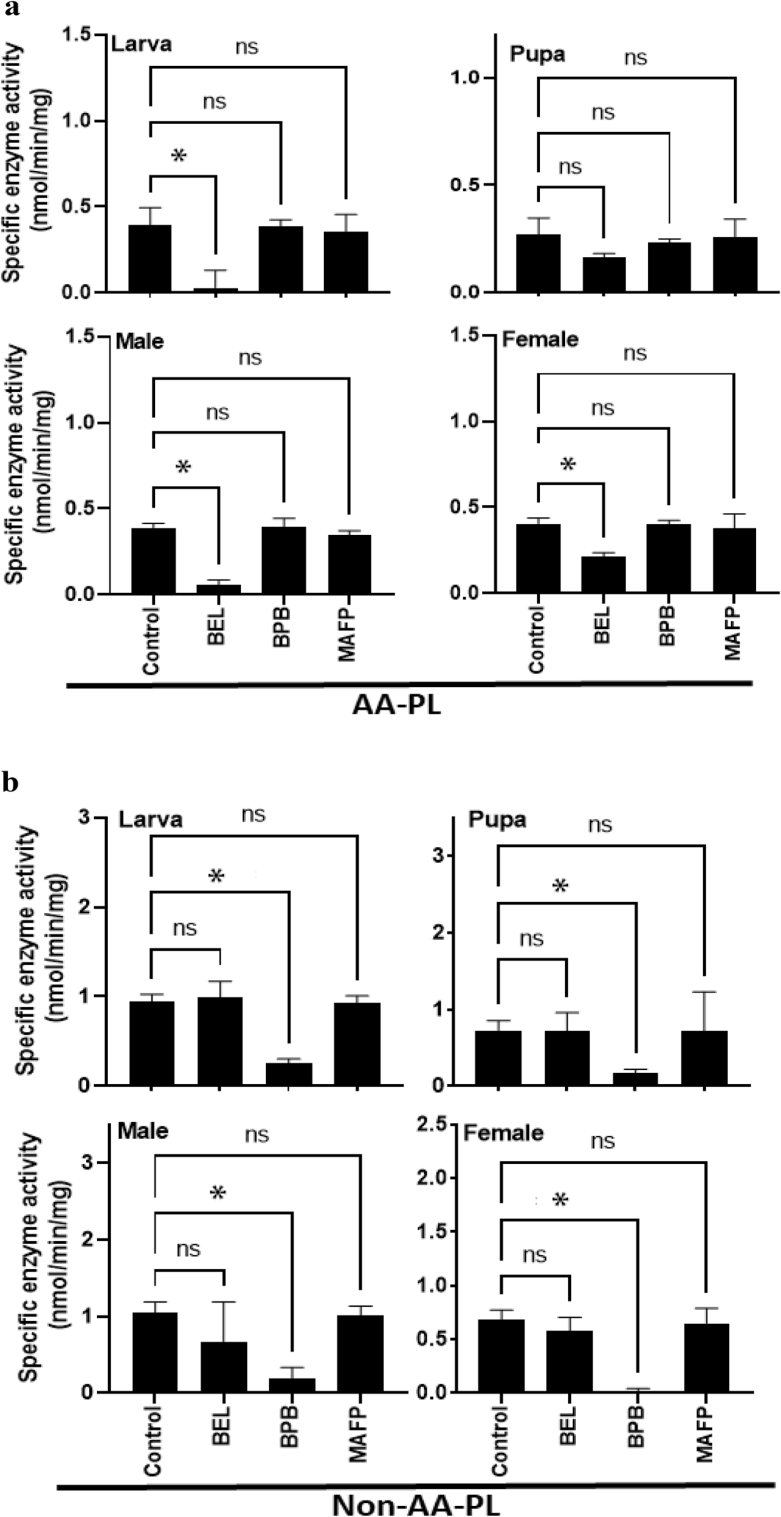
Variation of PLA_2_ activities in response to specific inhibitors in *F. occidentalis*: bromoenol lactone (‘BEL’, iPLA_2_ inhibitor), methylarachidonyl fluorophosphate (‘MAFP’, cPLA_2_ inhibitor), and *p*-bromophenacyl bromide (‘BPB’, sPLA_2_ inhibitor). (**a**) Variation in PLA_2_ susceptibility to the inhibitors under arachidonate phospholipid (‘AA-PL’) substrate. (**b**) Variation in PLA_2_ susceptibility to the inhibitors under non-arachidonate phospholipid (‘Non-AA-PL’). Enzyme extract was pre-incubated with each inhibitor for 15 min and the residual enzyme activity was estimated at 25 °C and pH 8.0. All treatments were replicated three times. Asterisk (*) indicates a significant difference at Type I error = 0.05 (LSD test) compared to control. ‘ns’ represents no significant difference.

The differential enzyme activities among developmental stages suggested multiple PLA_2_s in *F. occidentalis*. To test this hypothesis, PLA_2_ inhibitors specific to different PLA_2_ types were applied to the enzyme extracts (Fig. 2). Under AA-PL substrate, PLA_2_ activities were significantly suppressed by BEL (a specific inhibitor of iPLA_2_) treatment in most developmental stages except pupae (Fig. 2a). However, BPB (a specific inhibitor of sPLA_2_) or MAFP (a specific inhibitor of cPLA_2_) did not significantly inhibit the enzyme activities. In contrast, PLA_2_ activities at non-AA-PL substrate (Fig. 2b) were significantly suppressed by BPB treatment in all developmental stages but not by BEL and MAFP. These findings suggested that *F. occidentalis* possesses multiple PLA_2_s exhibiting different types of enzyme kinetics.

### Four different PLA_2_ genes of *F. occidentalis* and their variation in molecular structure and expression profile

**Figure 3:**
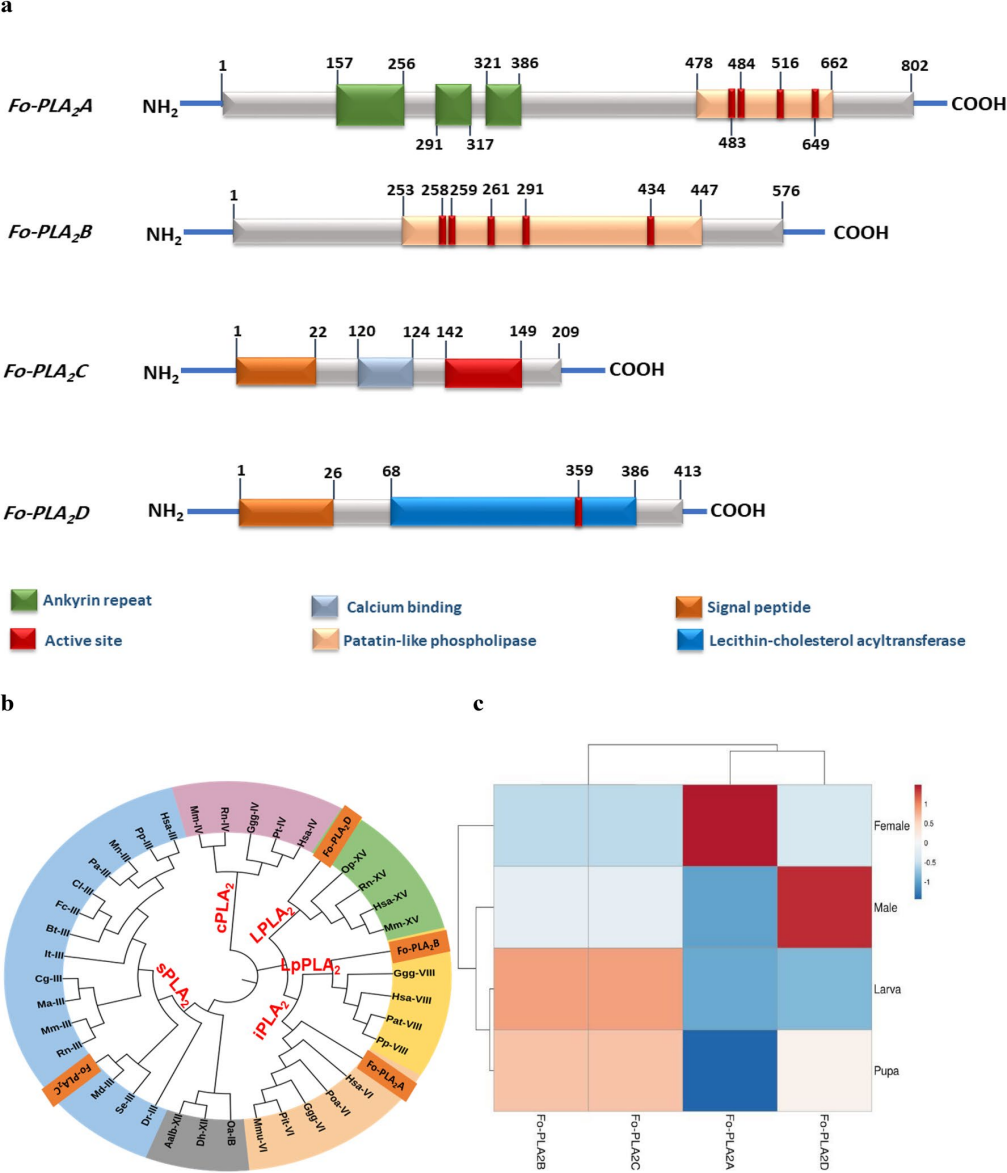
Four PLA_2_ genes (*Fo-PLA*_2_*A*,* Fo-PLA*_2_*B*,* Fo-PLA*_2_*C*,* Fo-PLA*_2_*D*) encoded in *F. occidentalis* genome and the variation in their expression profile. (**a**) Prediction of their functional domains using NCBI Conserved Domain Database (www. ncbi.nlm.nih.gov/cdd). (**b**) Classification of the four PLA_2_s with known PLA_2_s classified into secretory PLA_2_ (sPLA_2_: I, III, and XII), calcium-dependent cellular PLA_2_ (cPLA_2_: IV), calcium-independent cellular PLA_2_ (PLA_2_: VI), lipoprotein-associated PLA_2_ (Lp-PLA_2_: VIII), and lysosomal PLA_2_ (LPLA_2_: XV). This phylogenetic analysis was performed using MEGA6.06. Bootstrapping values were obtained with 1000 iterations to support branching and clustering. Amino acid sequences of PLA_2_s were retrieved from GenBank with accession numbers shown in Table S1. (**c**) Expression patterns of the four PLA_2_s in different developmental stages of *F. occidentalis*. The heatmap was generated and the pattern analysis by a phylogenetic tree was performed using the ClustVis online tool (https://biit.cs.us.ee/clustvis/).

Four PLA_2_ genes (*Fo-PLA*_2_*A*,* Fo-PLA*_2_*B*,* Fo-PLA*_2_*c*, and *Fo-PLA*_2_*D*) were encoded in the *F. occidentalis* genome (Fig. 3). Functional domain analysis indicated that all four PLA_2_s have their own catalytic domains predicted to be ‘active site’, ‘patatin-like phospholipase’, or ‘lecithin-cholesterol acetyltransferase’ (Fig. 3a). In addition, two PLA_2_s (*Fo-PLA*_2_*C* and *Fo-PLA*_2_*D*) are secretory due to the presence of signal peptide domain while the other two (*Fo-PLA*_2_*A* and *Fo-PLA*_2_*B*) are not. In the secretory types, *Fo-PLA*_2_*C* has a calcium-binding site, but *Fo-PLA*_2_*D* does not. In the non-secretory types, *Fo-PLA*_2_*A* has an ankyrin-repeat domain but *Fo-PLA*_2_*B* does not.

A phylogenetic analysis of the four PLA_2_s along with already identified groups (‘I-XVI’) of PLA_2_s (Fig. 3b) showed that they were separately clustered with group III (*Fo-PLA*_2_*C*), group VI (*Fo-PLA*_2_*A*), group VIII (*Fo-PLA*_2_*B*), and group XV (*Fo-PLA*_2_*D*). Traditionally, these groups are classified into secretory PLA_2_ (sPLA_2_) for group III, calcium-independent and intracellular PLA_2_ (iPLA_2_) for group VI, lipoprotein PLA_2_ (LpPLA_2_) for group VIII, and lysosomal PLA_2_ (LPLA_2_) for group XV.

These four different PLA_2_ genes were expressed in all developmental stages of *F. occidentalis* (Fig. 3c). However, their expression patterns varied among different stages. Analysis of these expression variations indicated a distinct difference between the expression patterns of the immature stages and those of adults. *Fo-PLA*_2_*A* and *Fo-PLA*_2_*D* showed a higher expression in adults while *Fo-PLA*_2_*B* and *Fo-PLA*_2_*C* showed a higher expression in immature stages. This distinct pattern was also supported by the phylogenetic pattern analysis.

### Fluorescence in situ hybridization (FISH) reveals tissue-specific PLA_2_s

**Figure 4:**
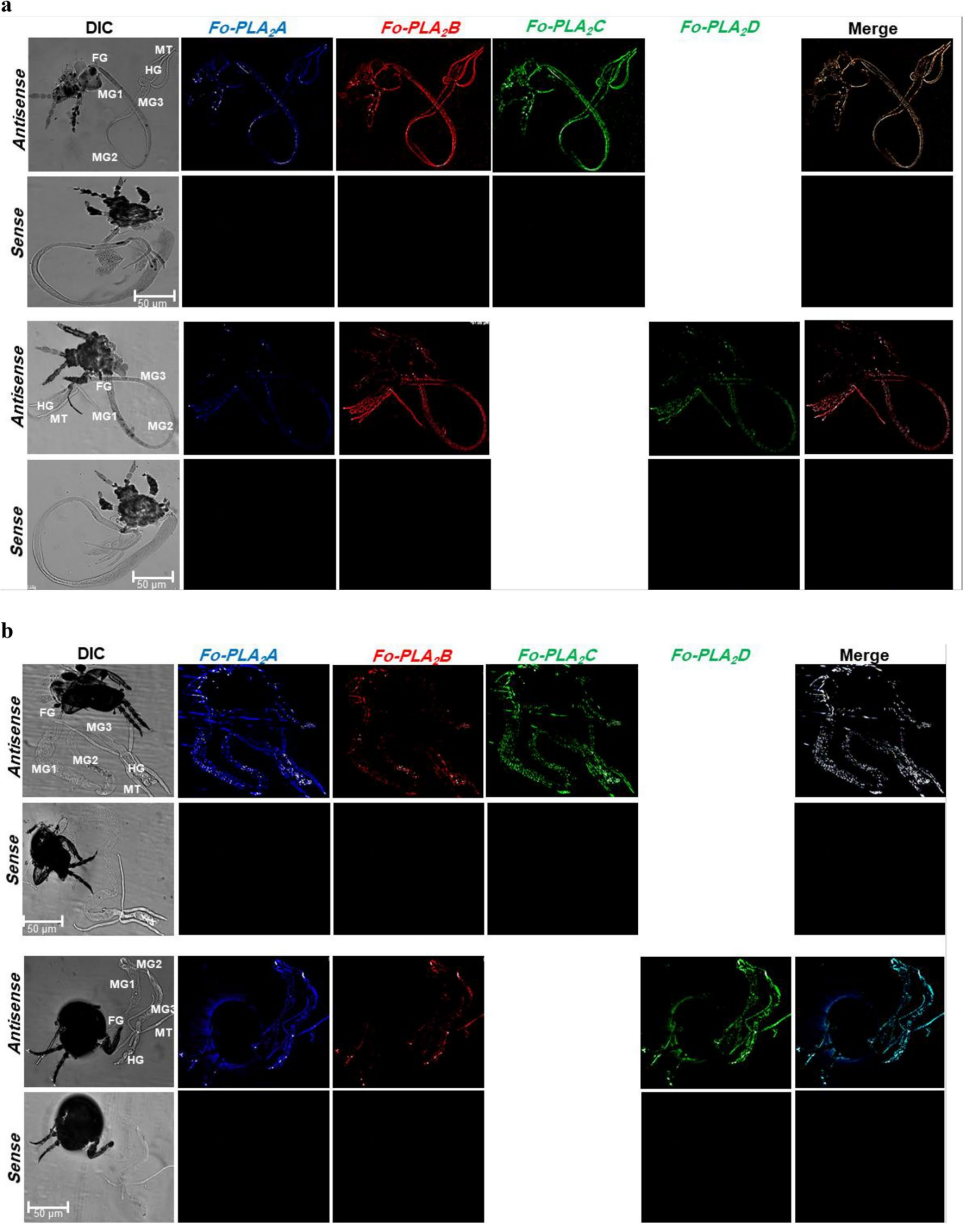

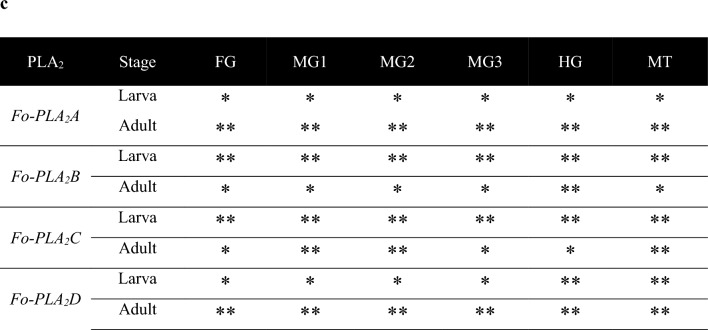
Variation in expression of four different PLA_2_ genes (*Fo-PLA*_2_*A*,* Fo-PLA*_2_*B*,* Fo-PLA*_2_*C*,* Fo-PLA*_2_*D*) in different larval (**a**) and adult (**b**) tissues of *F. occidentalis* by FISH analysis: foregut (FG), midgut (MG), hindgut (HG), Malpighian tubules (MT), and salivary gland (SG). Different fluorescence dyes (Marine blue, Rhodamine, and FITC) were used to monitor different PLA_2_s in FISH analyses, in which *Fo-PLA*_2_*C* and *Fo-PLA*_2_*D* were labeled by a common FITC and they were separately compared with other two PLA_2_s (*Fo-PLA*_2_*A* and *Fo-PLA*_2_*B*). Sense probes were used in each analysis as negative controls. (**c**) FISH signals in different tissues. The signals were categorized by the fluorescence intensity using ImageJ analysis (http://rsbweb.nih.gov/ij/): ** for > 2 and * for 0.1–2.

Intestines and salivary glands of both larvae (Fig. 4a) and adults (Fig. 4b) of *F. occidentalis* were examined for the expressions of the four PLA_2_ genes by performing FISH. The four PLA_2_ transcripts were specifically detected with their antisense probes but not with the sense probes, supporting the specificity of the FISH analysis. Even though all four PLA_2_ genes were expressed in larvae and adults, the FISH signals against *Fo-PLA*_2_*A* and *Fo-PLA*_2_*D* mRNAs were stronger in the intestinal organs of adults (Fig. 4c). In contrast, *Fo-PLA*_2_*B* and *Fo-PLA*_2_*C* mRNAs were highly expressed in the intestinal organs of larvae. This difference was evident in the different colors of the merged images, in which *Fo-PLA*_2_*A*+*Fo-PLA*_2_*B*+*Fo-PLA*_2_*D* showed a red color in larvae due to a relatively strong expression of *Fo-PLA*_2_*B* but blue-green color in adults due to a relatively strong expression of *Fo-PLA*_2_*D* while *Fo-PLA*_2_*A*+*Fo-PLA*_2_*B*+*FoPLA*_2_*C* showed a white color in both stages.

**Figure 5:**
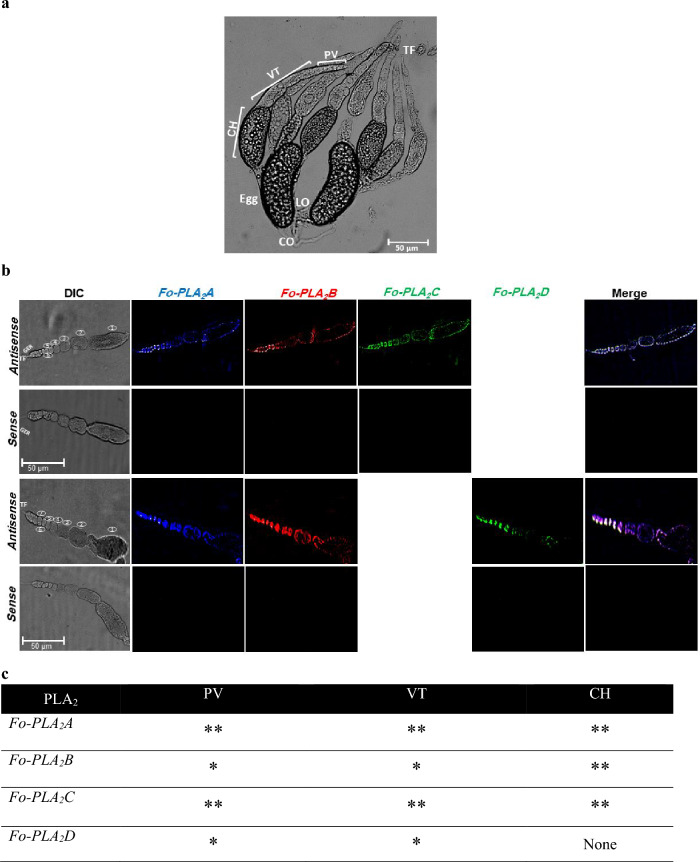
Variation in four different PLA_2_ genes in the ovary of *F. occidentalis*. Each ovariole was divided into previtellogenic (PV), vitellogenic (VT), and choriogenic (CH). (**a**) An ovary consisting of eight ovarioles along with lateral oviduct (LO), common oviduct (CO), and terminal filament (TF). (**b**) FISH analysis using different fluorescence dyes (Marine Blue, Rhodamine, and FITC) to monitor different PLA_2_s. Follicles in each ovariole are denoted by numbers in circle. Sense probes were used in each analysis as negative controls. (**c**) FISH signals in the ovary. The signals were categorized by the fluorescence intensity by using ImageJ analysis (http://rsbweb.nih.gov/ij/): ** for > 2, * for 0.1–2, and None for < 0.1.

In adult females, the four PLA_2_ mRNAs were examined in the ovary (Fig. 5). Four ovarioles were observed in each ovary, in which each ovariole was subdivided into previtellogenic (before formation of follicles), vitellogenic (growing oocytes in follicles), and choriogenic (terminal follicle in the ovariole undergoing chorion formation by follicular epithelium) regions (Fig. 5a). These ovarioles were tethered to the abdominal body wall through terminal filament. Some eggs were detected in the lateral oviduct by ovulation (see ‘egg’ in the oviduct). All four PLA_2_ genes were expressed in the ovary (Fig. 5b). Most PLA_2_s except *Fo-PLA*_2_*D* were expressed in the entire ovariole regions (Fig. 5c). *Fo-PLA*_2_*D* mRNA showed a low expression in the terminal ovariole area including the choriogenic follicle.

### PLA_2_s associated with immature development

Multiple PLA_2_s and their differential expressions suggested that they mediate different physiological processes in *F. occidentalis*. To test this hypothesis, two different loss-of-function experiments were devised. One approach was to suppress specific gene expressions by using individual RNAi treatments specific to each of the four PLA_2_ genes. The other approach entailed the use of specific PLA_2_ inhibitors to suppress the enzyme activities of specific PLA_2_s.

**Figure 6:**
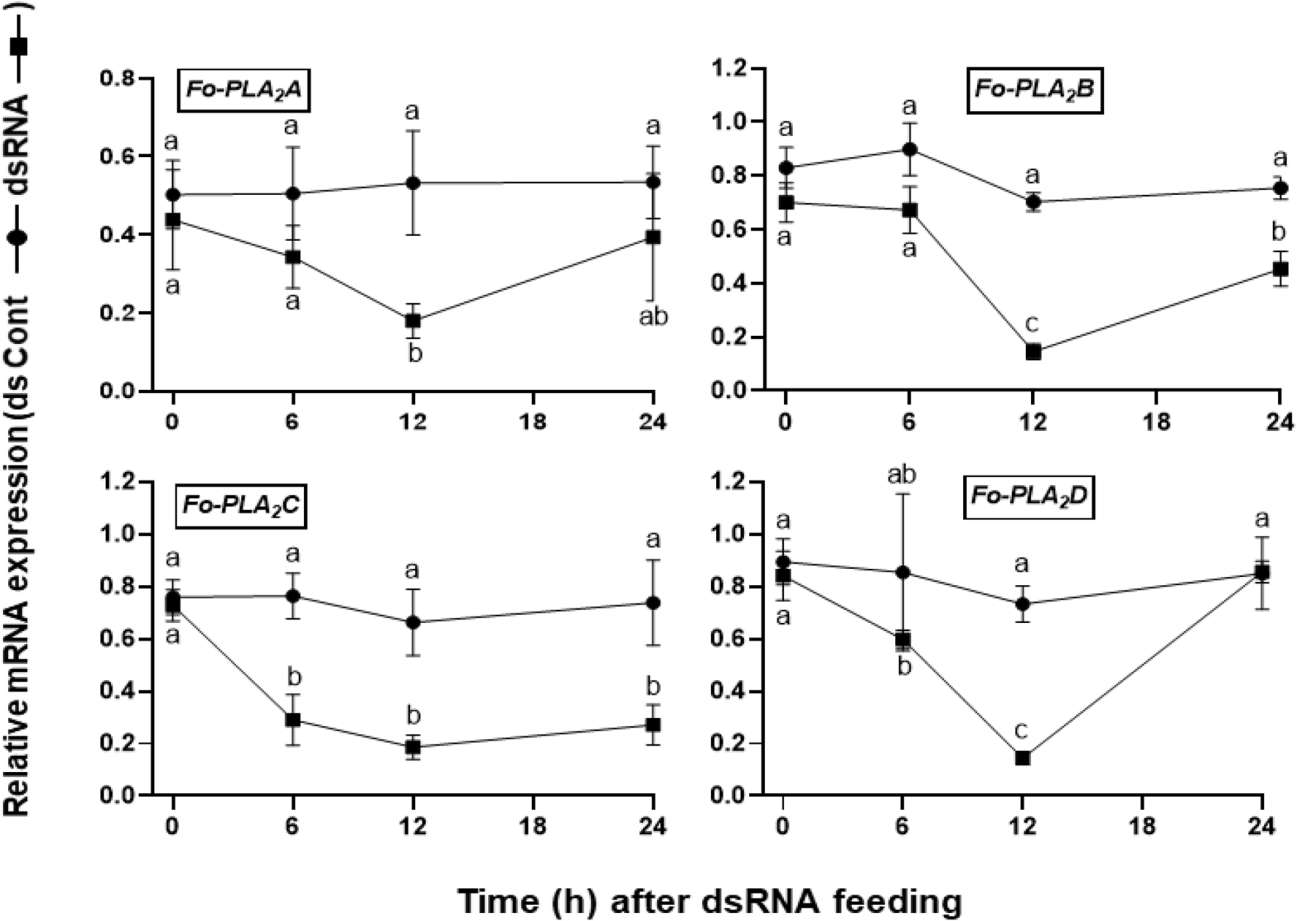
Individual RNAi treatments specific to each of four PLA_2_ genes (*Fo-PLA*_2_*A*,* Fo-PLA*_2_*B*,* Fo-PLA*_2_*C*,* Fo-PLA*_2_*D*) in *F. occidentalis*. RNAi was performed by feeding dsRNA specific to each PLA_2_ gene. A viral gene, *CpBV*302, was used as a control dsRNA (dsCON). An elongation factor, *EF*1, was used to normalize the expression level. Three replications were used per treatment. Different letters above standard deviation bars indicate significant difference among means at Type I error = 0.05 (LSD test).

**Figure 7:**
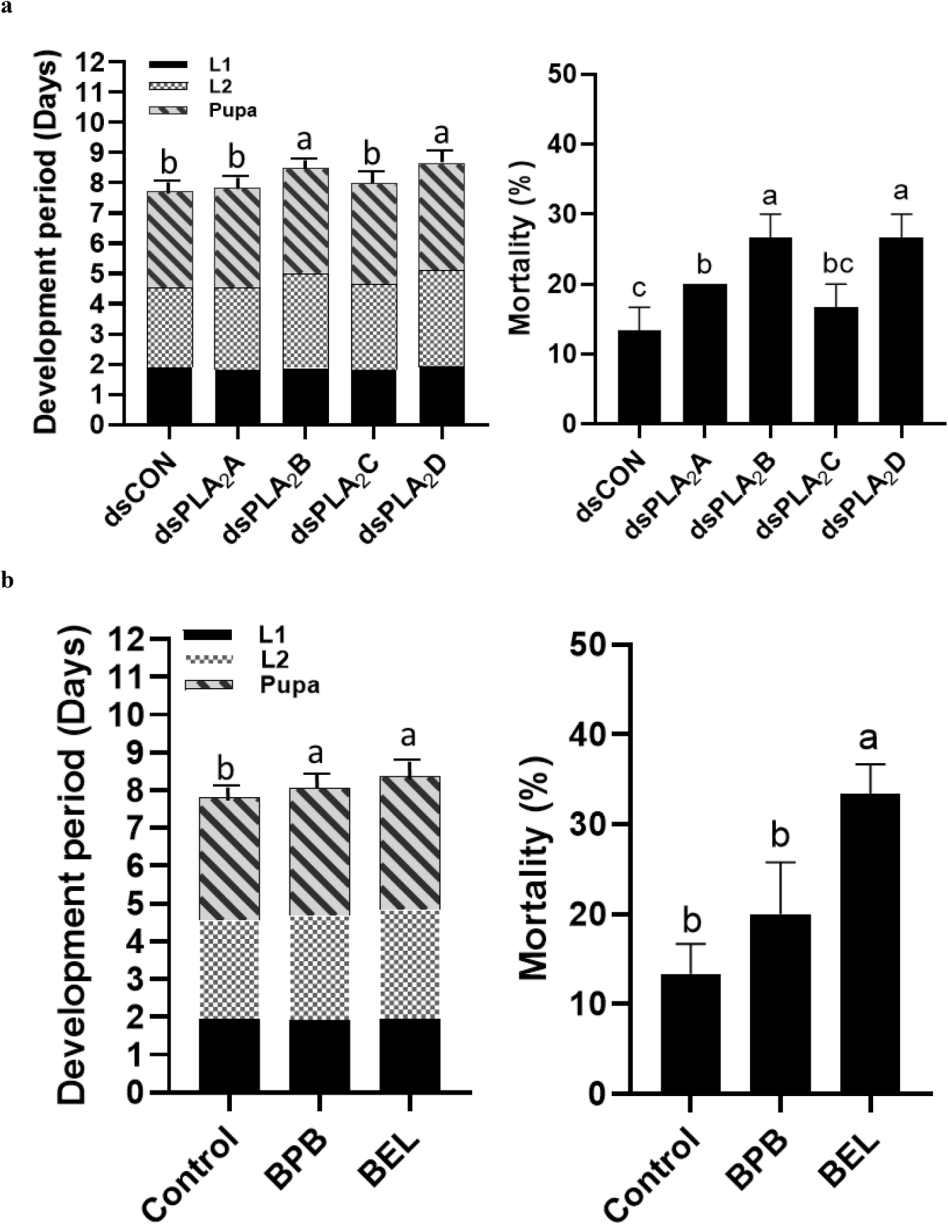
Differential influence of four PLA_2_s on immature development of *F. occidentalis*, in which immature stages include first (L1)/second instar (L2) larva and pupa. (**a**) Influence of individual RNAi treatments (dsPLA_2_A, dsPLA_2_B, dsPLA_2_C, dsPLA_2_D) of four PLA_2_ gene expressions on developmental period (left panel) and mortality (right panel). (**b**) Effect of PLA_2_ inhibitors (BEL and BPB) on developmental period (left panel) and mortality (right panel). Developmental period and mortality were assessed with 30 individuals as an experimental unit. Each treatment was replicated three times. Different letters above standard deviation bars indicate significant differences among means at Type I error = 0.05 (LSD test).

Individual RNAi against each of the four PLA_2_ genes was performed using a feeding method with specific dsRNAs (Fig. 6). These RNAi treatments resulted in more than 50% reduction in their target genes while RNAi control specific to nontarget genes did not influence the target gene expressions. Under these RNAi conditions, the development of immature stages of *F. occidentalis* was monitored (Fig. 7). Two RNAi treatments specific to *Fo-PLA*_2_*B* and *Fo-PLA*_2_*D* expressions led to significant developmental retardation in the immature stages, which resulted in significant mortality (Fig. 7a). However, the other two RNAi treatments specific to *Fo-PLA*_2_*A* and *Fo-PLA*_2_*C* expressions showed no influence on the development of the immature stages.

The larvae were treated with two PLA_2_ inhibitors (BEL and BPB) via the feeding method (Fig. 7b). Treatment with both inhibitors led to significant developmental retardation of the immature stages, and BEL treatment resulted in significantly higher mortality.

### PLA_2_s associated with immunity

**Figure 8:**
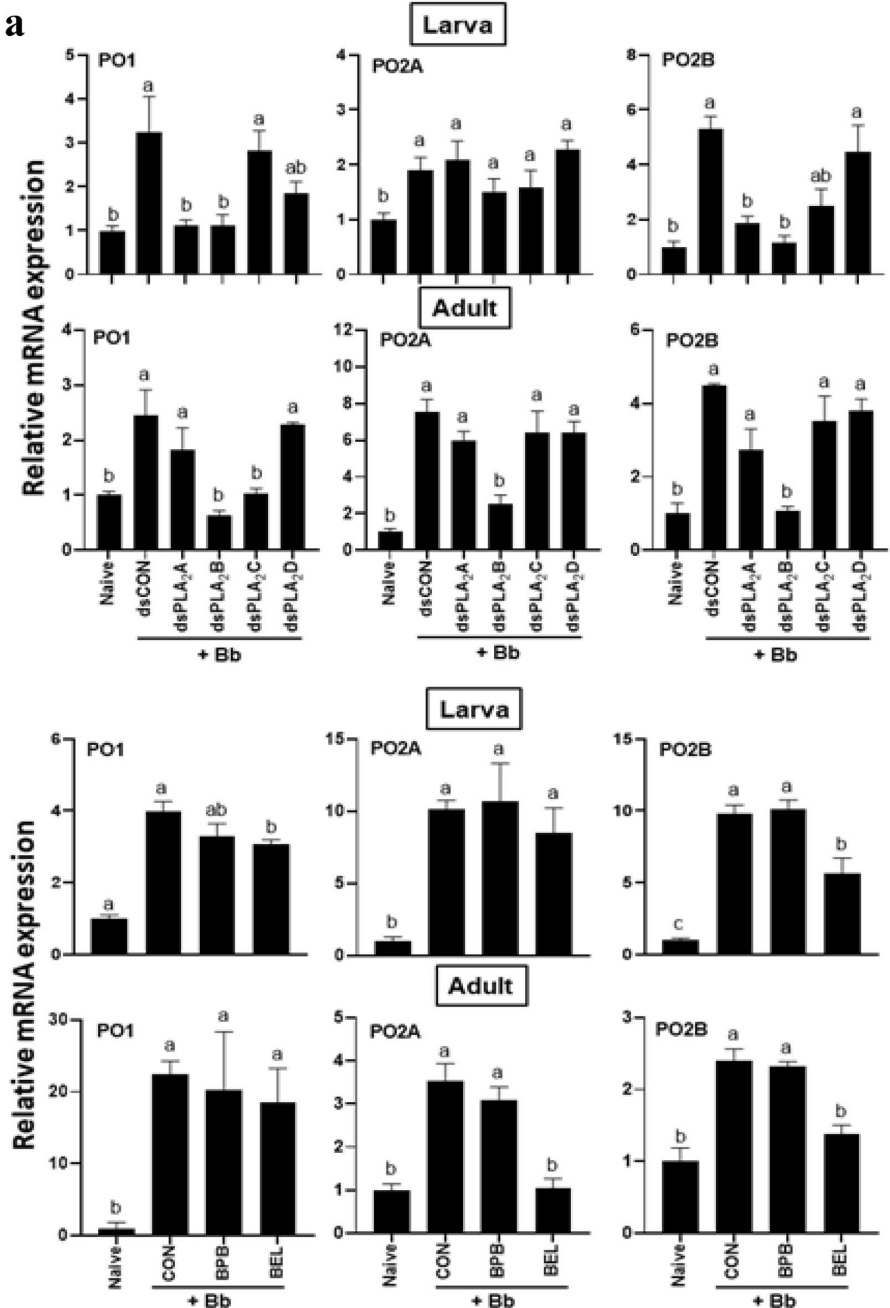

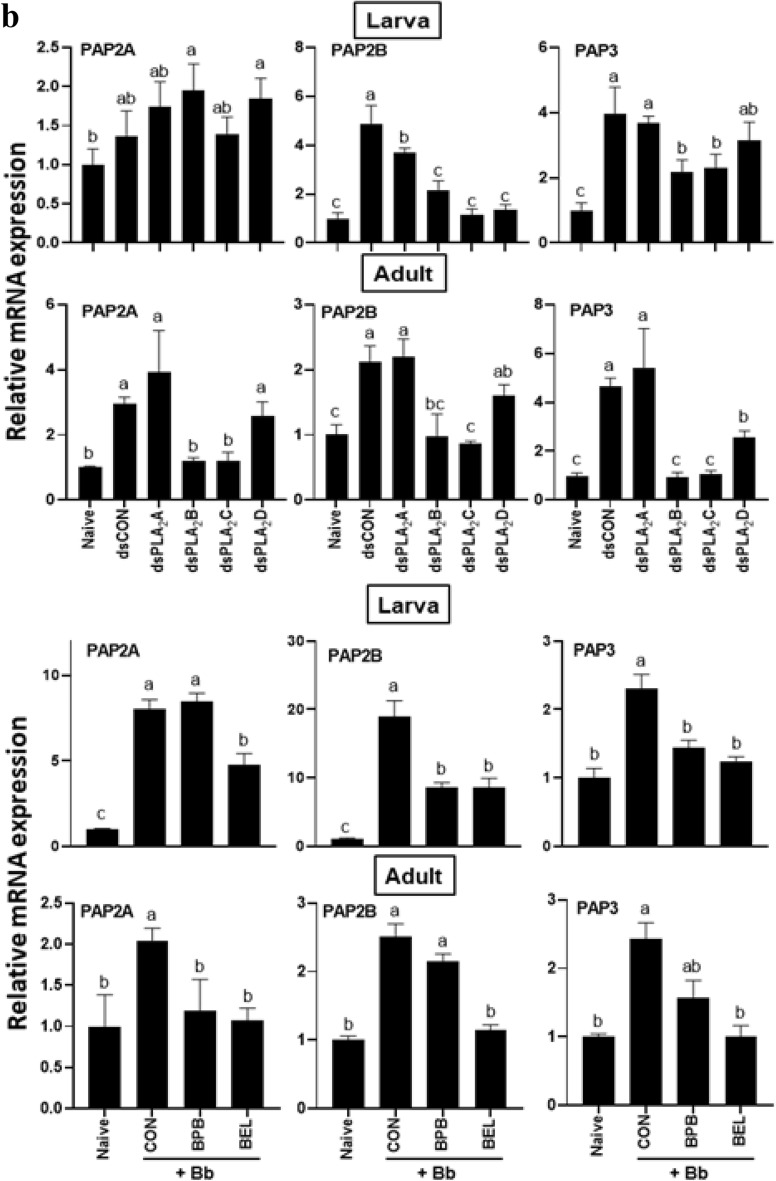

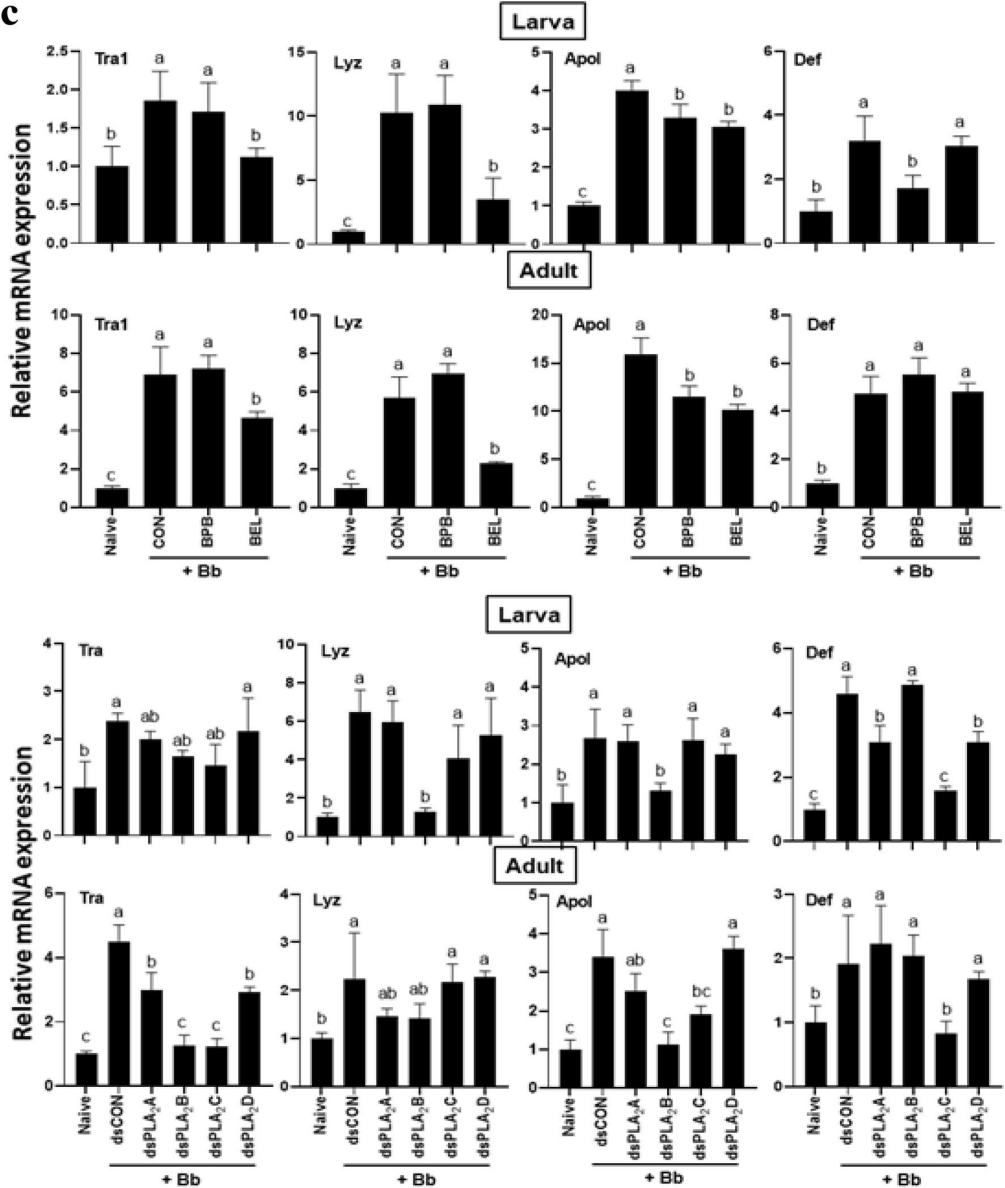
Differential influence of four PLA_2_s on the expression of immune-associated genes in larvae and adults of *F. occidentalis*. Influence of individual RNAi treatments (dsPLA_2_A, dsPLA_2_B, dsPLA_2_C, dsPLA_2_D, upper panels) of four PLA_2_ expressions or PLA_2_ inhibitors (BEL and BPB, lower panels) on expression of (**a**) three phenoloxidase genes (*PO*1, *PO*2*A*, and *PO*2*B*), (**b**) three prophenoloxidase-activating proteinase genes (*PAP*2*A*, *PAP*2*B*, and *PAP*3), and (**c**) four antimicrobial peptide genes (*Tra*1 for transferrin 1, *Lyz* for lysozyme, *Apol* for apolipophorin III, and *Def* for defensin). Immune challenge used *B. bassiana* by LC_50_ for each stage. Controls used dsRNA control for RNAi treatments or ethanol solvent for inhibitor treatments. A viral gene, *CpBV*302, was used as a control dsRNA (dsCON). An elongation factor, *EF*1, was used to normalize the expression level. In each treatment, total RNA was collected from the whole body extracts of ~ 100 larvae or ~ 100 adults after 18 h post-infection. Each measurement was replicated three times. Different letters above standard error bars indicate significant differences among means at Type I error = 0.05 (LSD test).

An immune challenge with an entomopathogenic fungus, *B. bassiana*, significantly up-regulated the expressions of three phenoloxidase (PO) genes (*PO*1, *PO*2*A*, and *PO*2*B*) of *F. occidentalis* in both larvae and adults (Fig. 8). However, individual RNAi treatments specific to most PLA_2_ genes of *F. occidentalis* significantly prevented the induction of the PO genes except for RNAi treatment specific to *Fo-PLA*_2_*D* expression (Fig. 8a). Especially, RNAi treatment specific *Fo-PLA*_2_*B* expression significantly inhibited the gene induction in both larvae and adults except for *PO*2*A* of larvae. Among the two specific inhibitors, BEL treatment suppressed the induction of PO gene expressions while BPB treatment did not.

The fungal infection also significantly up-regulated the expressions of three PO-activating proteinase (PAP) genes (*PAP*2*A*, *PAP*2*B*, and *PAP*3) in both larvae and adults (Fig. 8b). This induction was significantly prevented by RNAi treatments against different Fo-PLA_2_ genes. In particular, RNAi treatments specific to *Fo-PLA*_2_*B* or *Fo-PLA*_2_*C* expression significantly inhibited the induction of three PAP genes except for *PAP*2*A* at the larval stage. Two specific PLA_2_ inhibitors also prevented the gene induction in response to the fungal infection. BEL treatment suppressed the induction of all three PAP gene expressions while BPB treatment inhibited PAP genes depending on the developmental stage.

The expressions of four different AMP genes were up-regulated in response to fungal infection (Fig. 8c). However, the gene inductions were significantly suppressed by at least one of RNAi treatments specific to four PLA_2_ genes except for transferrin gene expression at the larval stage. RNAi treatment specific to *Fo-PLA*_2_*B* expression prevented the up-regulation of two AMP genes in both developmental stages. RNAi treatment specific to *Fo-PLA*_2_*C* expression prevented the up-regulation of three AMP genes in adults. Both PLA_2_ inhibitors also prevented the gene induction of the AMPs in response to the fungal infection. BEL treatment suppressed the induction of most AMP gene expressions except defensin. However, BPB treatment inhibited the induction of defensin at the larval stage. All these loss-of-function assays against AMP expressions suggest that specific PLA_2_s mediate PO gene expression in response to the immune challenge.

**Figure 9:**
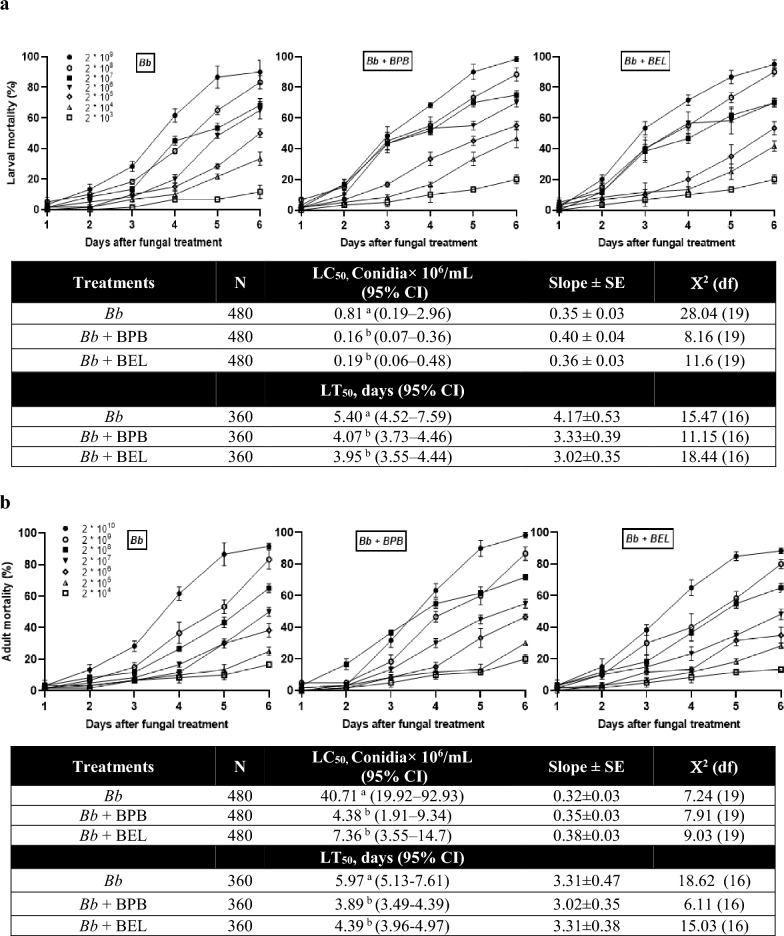
Influence of PLA_2_ on the anti-fungal response of *F. occidentalis* against *B. bassiana* in larvae (**a**) and adults (**b**). To inhibit PLA_2_ activity, two different PLA_2_ inhibitors were used to assess the change in fungal virulence. To estimate median lethal concentration (LC_50_) and time (LT_50_), a fungal concentration of 2 × 10^6^ conidia/mL for larvae or 2 × 10^8^ conidia/mL for adults was used. An experimental unit (= Petri dish) contained 20 larvae or adults. Each treatment was replicated three times. Different letters following median values indicate significant differences among means with non-overlapping in 95% confidence interval (CI).

To confirm the physiological roles of these PLA_2_s in immunity, the fungal virulence against larvae and adults of *F. occidentalis* was monitored after treatment with PLA_2_ inhibitors (Fig. 9). Fungal virulence was different in different developmental stages. Adults were more tolerant than larvae with > 50-fold higher median lethal concentration (LC_50_) though there was little difference in median lethal time (LT_50_). Both inhibitors of BPB and BEL significantly enhanced the fungal virulence against the thrips in larvae and adults, which led to significant decreases in lethal dose (= LC_50_) and speed-to-kill (= LT_50_).

### PLA_2_s associated with reproduction

**Figure 10:**
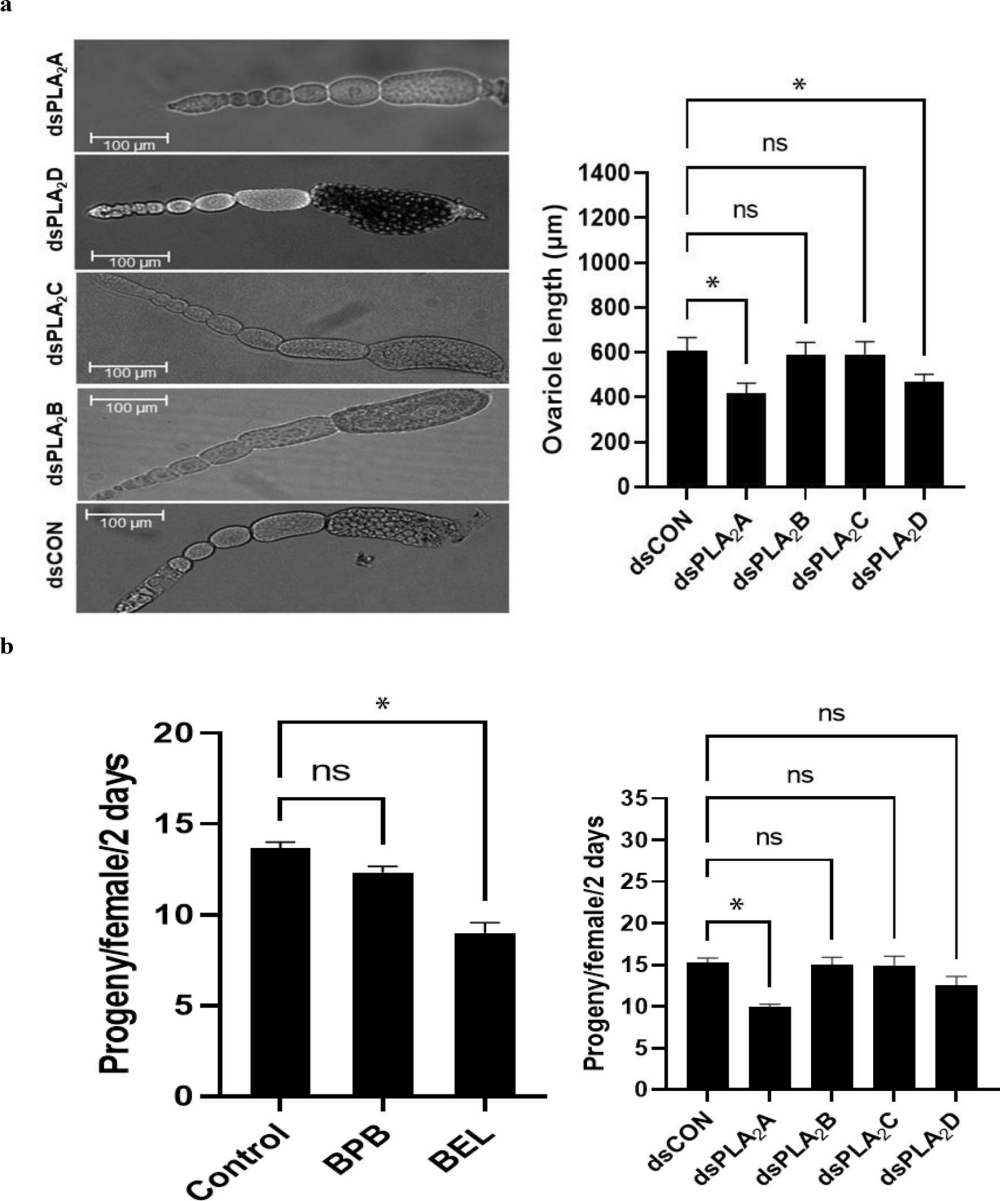
Differential influence of four PLA_2_s on reproductive processes of *F. occidentalis*. Influence of individual RNAi treatments (dsPLA_2_A, dsPLA_2_B, dsPLA_2_C, dsPLA_2_D) of four PLA_2_ expressions or PLA_2_ inhibitors (BEL and BPB) on ovariole development (**a**) and fecundity (**b**). Ovariole development was assessed in 10 adults by measuring the entire ovariole length. For the fecundity test, 10 females comprised an experimental unit, in which progeny number laid for two days was counted. Each treatment was replicated three times. A viral gene, *CpBV*302, was used as a control dsRNA (dsCON). Asterisk (*) indicates a significant difference at Type I error = 0.05 (LSD test) compared to control. ‘ns’ represents no significant difference.

To determine specific PLA_2_ gene(s) that mediate ovary development, individual RNAi treatments specific to each of the four PLA_2_ genes were applied to *F. occidentalis* (Fig. 10). Two RNAi treatments specific to *Fo-PLA*_2_*A* or *Fo-PLA*_2_*D* expression led to a significant reduction in ovariole development while RNAi treatments specific to *Fo-PLA*_2_*B* or *Fo-PLA*_2_*C* expression did not (Fig. 10a). Especially, the RNAi treatment specific to *Fo-PLA*_2_*A* expression resulted in a significant decrease in fecundity (Fig. 10b). Reduced fecundity was also observed in the females treated with BEL, but not in those treated with BPB.

## Discussion

Transcriptome analyses suggested that eicosanoids mediate the immune responses of *F. occidentalis* against viral or fungal infections^23,24^. However, the biosynthetic activity of the eicosanoids in the thrips against pathogen infections is not well characterized. To better understand the roles of eicosanoids in the thrips, this study focused on PLA_2_, which catalyzes the committed step in the biosynthesis of eicosanoids by assessing its biochemical characteristics and its catalytic modulation upon immune challenge. In particular, this study analyzed the independent roles of the four different PLA_2_s encoded in *F. occidentalis* by assessing the development and reproduction after individual RNAi treatments.

Four PLA_2_ genes are encoded and expressed in *F. occidentalis*. In addition to RT-qPCR, FISH was performed to determine their expressions in larval midgut and adult ovaries. They are classified into sPLA_2_ and iPLA_2_. All four PLA_2_s were expressed in larvae and adults of thrips. PLA_2_ have been found in all biological systems from bacteria to humans and are classified into at least 16 groups (I–XIV) based on their amino acid sequences^8^. These diverse PLA_2_s are divided into sPLA_2_, iPLA_2_ (Ca^2+^-independent cellular PLA_2_), and cPLA_2_ (Ca^2+^-dependent cellular PLA_2_). Groups III (Fo-sPLA_2_C) and XV (Fo-sPLA_2_D) are sPLA_2_s whereas groups VI (Fo-PLA_2_A) and VIII (Fo-PLA_2_B) are iPLA_2_s. No cPLA_2_ was encoded in the *F. occidentalis* genome like in other insects. Most insect sPLA_2_s are classified into group III, which are divided into venomous and non-venomous PLA_2_s^8^. This study identified a novel sPLA_2_ (= Fo-sPLA_2_D) in insects, which was classified into group XV. This type of PLA_2_ was first reported in a cnidarian invertebrate, *Adamsia carciniopados*, and has been regarded as an ancient PLA_2_ prototype^25^. Two different types of group VI PLA_2_s are found in insects and classified based on the presence of ankyrin repeat domains^8^. Fo-PLA_2_A has three ankyrin domains and is classified into ankyrin type of iPLA_2_. In contrast, Fo-PLA_2_B is a novel insect PLA_2_ classified into group VIII. The catalytic activity of group VIII PLA_2_s is known to be Ca^++^-independent and they display platelet-activating factor acetylhydrolase activity^2^. Thus, this study reports two novel insect PLA_2_s in *F. occidentalis* classified into groups VIII and XV.

All four Fo-PLA_2_ genes were expressed in different developmental stages. This explains the detection of PLA_2_ activities in all developmental stages. However, the PLA_2_ activities measured with two different substrates varied among developmental stages. Based on K_M_ values indicating substrate affinity of the PLA_2_, adults preferred AA-linked phospholipid while larvae preferred non-AA phospholipid. This suggests that the different developmental stages of the thrips possess different compositions of the four PLA_2_s because cellular PLAs prefer AA type while sPLA_2_s do not have preference^2^. The different PLA_2_ compositions among the developmental stages were supported by the differential susceptibilities to different inhibitors. First, MAFP which is a specific inhibitor of cPLA_2_ activity^26^ did not inhibit the PLA_2_ activities in all the developmental stages. Second, BEL, a specific inhibitor of iPLA_2_^27^ inhibited the enzyme activities only in AA type substrate, in which it significantly inhibited the enzyme activities of larvae and adults, but not that of pupae. Third, BPB a specific inhibitor of sPLA_2_ activity^28^ inhibited the enzyme activities in all developmental stages only in non-AA type substrate. These findings suggest that different developmental stages of *F. occidentalis* possess different PLA_2_ activities in terms of catalytic activity and substrate preference probably by modulating differential expression of the four PLA_2_ genes to mediate the specific physiological processes. This differential expression of the four PLA_2_ genes may explain the relative preference for AA-PL in male adults and non-AA-PL in larvae measured by K_M_ values.

Inhibition of PLA_2_ activity led to developmental retardation of *F. occidentalis*. Both BPB and BEL, which are inhibitors of PLA_2_ activities of *F. occidentalis*, significantly interfered with the immature development of the thrips. Individual RNAi treatments specific to each of the PLA_2_ genes also resulted in developmental retardation, in which suppression of *Fo-PLA*_2_*C* expression was the most effective. sPLA_2_ is secreted from the midgut and plays a crucial role in digesting dietary lipids in *Manduca sexta*^29,30^. The mechanism of lipid digestion in insects is controversial due to the lack of bile salts to solubilize dietary lipids. According to a hypothesis, sPLA_2_ provides lysophospholipid (LPL) from dietary phospholipids to act like insect bile salts^1^. sPLA_2_ of *S. exigua* secreted from the midgut was specifically inhibited by benzylideneacetone (BZA), a specific PLA_2_ inhibitor, by feeding to larvae, which led to a significant decrease in gut content sPLA_2_ activity, body growth, and total hemolymph lipid contents^31^. However, the addition of a specific LPL, 1-palmitoyl-sn-glycero-3-phosphocholine, to BZA-treated larvae significantly rescued the digestibility and subsequent larval growth^31^. These findings explain the developmental retardation of the thrips by the suppression of PLA_2_ activities and suggest a digestive role of Fo-PLA_2_C in the thrips.

PLA_2_ activity of *F. occidentalis* was associated with immune responses and its suppression increased the susceptibility to a fungal pathogen, *B. bassiana*. Both BEL and BPB significantly enhanced the fungal virulence to the thrips in both larvae and adults. This supports a previous study^23^ which demonstrated a crucial role of eicosanoid-mediated immune responses in defending fungal pathogenicity. A pertinent question is which of the four PLA_2_(s) mediates the immune responses in *F. occidentalis*. Individual RNAi treatments showed that all four PLA_2_s mediated the gene expressions associated with AMPs, melanization, or production of reactive oxygen species. The regulation of the expression of immune-associated genes by the PLA_2_ activities may be performed by eicosanoids synthesized from the PLA_2_ catalytic activity^32^. A transcriptional factor called Repat33 is a downstream component of the eicosanoid immune signaling pathway in *S. exigua*, in which Repat33 mediates the immune-associated gene expression and cellular immune response^33^. In addition, prostaglandin E_2_ (PGE_2_) receptors have been identified in lepidopteran and dipteran insects and their downstream signals up-regulate cAMP and Ca^2+^ levels^34–36^. Thus, the elevated PLA_2_ activity upon immune challenge upregulates PLA_2_ activity, which produces eicosanoids to mediate the expressions of the immune-associated genes. In particular, Fo-PLA_2_B and Fo-PLA_2_C may play crucial roles in mediating various immune responses in *F. occidentalis* because their RNAi treatments mostly interfered with the immune-associated genes.

Oocyte development in *F. occidentalis* was found to be dependent on PLA_2_ activity. In particular, iPLA_2_ activity played a crucial role in oocyte development because treatment with BEL but not BPB inhibited oocyte development. Furthermore, in the individual RNAi treatments, suppression of *Fo-PLA*_2_*A* expression significantly inhibited oocyte development. In addition, RT-qPCR and FISH analysis showed high expression of *Fo-PLA*_2_*A* in adults. In *Drosophila melanogaster* and *S. exigua*, PGE_2_ was shown to mediate nurse cell dumping during early vitellogenesis in the polytrophic ovarioles^37,38^. In *F. occidentalis*, PGE_2_ was detected in growing ovarian follicles by an immunofluorescence assay, and aspirin (a specific cyclooxygenase inhibitor) treatment significantly suppressed the oocyte development in previtellogenesis and choriogenesis^39^. The present study suggests that Fo-PLA_2_A may play a crucial role in the production of PGE_2_ in the ovary to facilitate the oocyte development in *F. occidentalis*.

These results suggest that the four Fo-PLA_2_s are required for mediating development, immunity, and reproduction with their unique enzyme activities and differential expressions in different developmental stages and tissues of *F. occidentalis*. Specifically, our inhibitor assays coupled with individual RNAi treatments suggest that Fo-PLA_2_C mediates physiological processes especially in development, Fo-PLA_2_A in reproduction, and Fo-PLA_2_B/C in immunity though all four PLA_2_s are associated with the different physiological processes.

## Experimental procedures

### Thrips rearing and fungal culture

Adult *F. occidentalis* were collected from a hot pepper field in Andong, Korea, and reared on sprouted bean seed kernels under controlled laboratory conditions: a constant temperature of 27 ± 1 °C, photoperiod of 16:8 h (L:D), and relative humidity (RH) of 60 ± 5%. Under these conditions, the thrips underwent three larval stages (L1-L3), prepupa, and pupa before the emergence of adult. L2 stage and young adults (< 3 days after adult emergence) were used for pathogenicity tests. *B. bassiana*, an entomopathogenic fungus, was cultured for 14 days in a potato dextrose agar (PDA) plate at 25 ± 1 °C, 70 ± 5% relative humidity, and a 16:8 h (L:D) photoperiod.

### Chemicals

PLA_2_ assay kits and methyl arachidonyl fluorophosphonate (MAFP) were purchased from Cayman Chemical (Ann Arbor, MI, USA). *p*-Bromophenacyl bromide (BPB), bromoenol lactone (BEL), bovine serum albumin (BSA), dimethylsulfoxide (DMSO), and t-octylphenoxy-polyethoxyethanol (Triton X-100) were purchased from Sigma Aldrich Korea (Seoul, Korea). Phosphate-buffered saline (PBS) was prepared with 100 mM phosphoric acid. Its pH was calibrated to 7.4 using 1 N NaOH.

### PLA_2_ enzymatic activity

PLA_2_ activities in whole bodies of 100 individuals at each stage (L2 larva, pupa, and adult) were measured using sPLA_2_ and cPLA_2_ assay kits (Cayman Chemical) containing arachidonyl thio-phosphatidyl choline (PC) and diheptanoyl thio-PC substrates, respectively, based on the method described by Vatanparast et al.^7^. Whole body extracts were obtained after homogenizing in PBS. Moreover, to assess specific PLA_2_ inhibitors (BPB, BEL, and MAFP) were pre-incubated at 400 µM final concentration with each enzyme for 15 min, and residual enzyme activities were measured at 25 °C. All treatments were replicated three times. Protein concentration was determined by Bradford^40^ assay using BSA as standard.

### Bioinformatics and phylogenetic analyses of PLA_2_s

Phospholipase A_2_ sequences (*Fo-PLA*_2_*A*, *Fo-PLA*_2_*B*, *Fo-PLA*_2_*C*, and *Fo-PLA*_2_*D*) of *F. occidentalis* were obtained from the National Center for Biotechnology Information (www.ncbi.nlm.nih.gov) with accession numbers of XM_026421156.2, XM_026433123, XM_026415753, and XM_026437429, respectively. Phylogenetic analyses were performed using MEGA6.06 and ClustalW programs from EMBL-EBI (www.ebi.ac.uk). Bootstrapping values were obtained with 1000 iterations to support branching and clustering. Conserved domains of the four PLA_2_s were predicted using the NCBI Conserved Domain Database (www. ncbi.nlm.nih.gov/cdd).

### FISH assay

To localize the four PLA_2_s in different tissues, the larval guts and adult ovaries were isolated onto a sterilized glass slide and fixed with 4% paraformaldehyde for 1 h at room temperature (RT). After washing with PBS, the tissues were permeabilized with 1% Triton X-100 in PBS for 2 h at RT. After washing with PBS, the tissues were rinsed in 2 × sodium saline citrate (SSC) and incubated at 42 °C with 25 μL of pre-hybridization buffer (2 μL yeast tRNA, 2 μL 20 × SSC, 4 μL dextran sulfate, 2.5 μL 10% SDS, and 14.5 μL deionized H_2_O) in dark and humid conditions for 1 h. Then, the pre-hybridization buffer was replaced with hybridization buffer (5 μL deionized formamide and 1 μL fluorescein-labeled oligonucleotide in 19 μL of the pre-hybridization buffer). DNA oligonucleotide probes were labeled at the 5′ ends with fluorescein amidite (FAM), rhodamine, or marina blue, which were purified using high-performance liquid chromatography (Bioneer, Daejeon, Korea). The probe sequences are listed in Table S1. The slides were covered with an RNAse-free cover slip and kept overnight (16–17 h) in a humid chamber at 42 °C. After hybridization, the tissues were washed twice with 4 × SSC for 10 min each and incubated with 4 × SSC containing 1% Triton X-100 in RT for 5 min. After washing three times with 4 × SSC, tissue samples were incubated at 37 °C with 1% anti-rabbit antibody (Thermo Fisher Scientific, Wilmington, DE, USA) in PBS under dark conditions for 30 min. After incubation, the tissues were washed twice with 4 × SSC for 10 min each, once with 2 × SSC, and then allowed to dry in air. After adding a drop of 50% glycerol and incubating at RT for 15 min, samples were covered by cover slip, and the slides were observed under a fluorescence microscope (DM2500, Leica, Wetzlar, Germany).

### RNA extraction and RT-qPCR

Total RNA was extracted from different developmental stages of *F. occidentalis* using Trizol reagent (Invitrogen, Carlsbad, CA, USA) according to the manufacturer’s instructions. An experimental unit consisted of approximately 100 larvae, pupae, and adults. The extracted RNAs were quantified using a spectrophotometer (NanoDrop, Thermo Fisher Scientific). RNA extract (100 ng per reaction) was used for cDNA synthesis with an RT-premix (Intron Biotechnology, Seoul, Korea). Quantitative PCR (qPCR) was performed using SYBR Green Real-Time PCR master mixture (Toyobo, Osaka, Japan) on a Real-Time PCR System (Step One Plus Real-Time PCR System, Applied Biosystems, Singapore). The reaction mixture (20 μL) contained 10 pmol of gene-specific primers (Table S2) used in RT-PCR and 80 ng of cDNA template. After activating Hotstart Taq DNA polymerase at 94 °C for 5 min, the reaction was amplified with 40 cycles of denaturation at 94 °C for 30 s, annealing at a specific temperature depending on primers (Table S2) for 30 s, and extension at 72 °C for 30 s. The target gene expression levels were normalized to those of *EF*1, a reference gene. Each treatment was replicated with three independently prepared biological samples. Quantitative analysis was performed using the comparative CT (2^−ΔΔCT^) method^41^.

### RNA interference (RNAi)

Double-stranded RNAs (dsRNAs) were used for RNAi. To prepare dsRNAs specific to different genes, template DNAs were amplified with forward and reverse gene-specific primers containing the T7 promoter sequence at their 5ʹ ends. The resulting T7 promoter-tagged template DNAs were used to construct dsRNAs using the MEGAscript RNAi kit (Ambion, Austin, TX, USA). The newly-formed dsRNAs were mixed with Metafectene PRO (Biontex, Plannegg, Germany), a transfection reagent, at a 1:1 (v/v) ratio, and incubated at 25 °C for 30 min to form liposomes. These dsRNAs were treated by the feeding delivery method. Briefly, the beans were soaked in a dsRNA suspension at 500 μg/mL for 20 min. After removing the excess moisture, the treated beans were placed in a circular breeding container (100 mm × 40 mm) for 24 h, accessible to *F. occidentalis* individuals. RNAi efficiency was evaluated at different time intervals by RT-qPCR. Each treatment was replicated three times.

### Effect of RNAi treatment on immature development

Specific dsRNAs were used to evaluate the different PLA_2_ functions in the immature developmental period. Within 6 h after the emergence of the first instar larvae, 10 newly emerged larvae were fed beans soaked in PLA_2_ dsRNAs. The treated diet was replaced every 24 h and the developmental stage period was measured every day till adult emergence. Three repetitions were performed for each treatment.

### Effect of RNAi treatment on reproductive processes

Within six hours of adult emergence, a group consisting of twelve females and two males were provided with beans soaked in the dsRNA suspensions for a continuous 36-h period, with the beans being replaced every 12 h. Subsequently, fresh untreated bean cotyledons were provided for 48 h to facilitate egg laying by the test thrips. The count of eggs laid by the females over two days was determined by observing the newly hatched larvae on the beans. Furthermore, the same treatment method was employed to evaluate ovary size. After treatment, the ovaries were dissected and isolated, and their size was measured. Each treatment was replicated three times.

### Effects of inhibitors (BPB and BEL) on immature development

To evaluate the impact of PLA_2_ inhibitors on the developmental period, 10 newly emerged first-instar larvae were provided with beans soaked in inhibitors (BEL and BPB) within 6 h of their emergence. The treated diet was refreshed every 24 h, and the developmental stages and mortality were monitored daily until adult emergence. This experiment was replicated three times for each treatment.

### Effects of inhibitors (BPB and BEL) on reproductive processes

Six hours after adult emergence, adult thrips were exposed to beans soaked in PLA_2_ inhibitors (BPB and BEL) for a continuous 36-h period, renewing the treated beans every 12 h. Subsequently, untreated fresh bean cotyledons were provided for 48 h to facilitate egg deposition. The count of eggs laid by the females over two days was determined by monitoring the emergence of newly hatched larvae on the beans. Each treatment involved 10 females and 2 males, and the experiment was replicated three times.

### Preparation of *B. bassiana *suspension and its virulence against *F. occidentalis*

Conidial suspension of *B. bassiana* was prepared by collecting the fungal colonies cultured on PDA medium in 1 mL of Triton X-100 (0.1%) (Duksan Pure Chemicals, Ansan, Korea) in PBS. Conidia of the suspension were counted using a Neuberger hemocytometer (Marienfeld-Superior, Lauda-Königshofen, Germany) under 40 × magnification.

To assess the virulence of *B. bassiana*, L2 larvae and adults were fed with different concentrations (1 × 10^4^, 1 × 10^5^, 1 × 10^6^, 1 × 10^7^, 1 × 10^8^, 1 × 10^9^ 1 × 10^10^ conidia/mL) of conidial suspension. Briefly, a piece of sprouted bean seed kernel was dipped in 1 mL of conidial suspension from each concentration for 5 min and kept for 10 min to dry under a clean bench. After L2 larvae or adults were released into a petri dish (5 × 2 cm), the dish was sealed with parafilm (Bemis Company, Zurich, Switzerland). These petri dishes were kept in a desiccator (4202-0000, Bel-Art Products, Pequannock, NJ, USA) with a constant temperature of 25 ± 1 °C and 75 ± 5% RH which was maintained using a saturated solution of NaCl according to Winston and Bates^42^. Dead insects were counted every 24 h up to 6 days by confirming mycosis development on insect cadavers. Three replicas of each treatment were used and each replicate used 20 insects.

### Data analysis

Analysis of variance (ANOVA) followed by post hoc Tukey's test were conducted for statistical analysis using GraphPad Prism version 8.2.0 (La Jolla, CA, USA). Bioassay data were used to estimate the median lethal concentration (LC_50_) and time (LT_50_) using PoloPlus^43^. Significant differences between LC_50_ values were determined as described by Robertson et al.^44^.

### Supplementary Information

Supplementary Information.

## Data Availability

All data generated or analyzed during this study are included in this published article and its supplementary information file. The genome sequence datasets generated and/or analyzed during the current study are available in the GenBank repository using accession numbers in Table S1.
