# Supplementary material for: Four phospholipase A2 genes encoded in the western flower thrips genome and their functional differentiation in mediating development and immunity
Source: Sci Rep. 2024 Apr 29;14:9766. doi: 10.1038/s41598-024-60522-8 (PMC11059263; doi:10.1038/s41598-024-60522-8)
Supplement: Supplementary file 1 — Supplementary Information. [file 41598_2024_60522_MOESM1_ESM.docx]

**Supplementary information**

**Table S1.** GenBank accession numbers of genes used in this study

**Table S2.** Primers used in this study

**Table S3.** Probe sequences used in FISH assay

**Table S1**. GenBank accession numbers of genes used in this study

| **Organism** | **Gene name** | **Acronym** | **Accession number** |
| --- | --- | --- | --- |
| *Bos taurus* | PLA_2_ | Bt-III | NP_001074379.2 |
| *Canis lupus* | PLA_2_ | Cl-III | XP_025330373 |
| *Cricetulus griseus* | PLA_2_ | Cg-III | XP_003509348 |
| *Danio rerio* | PLA_2_ | Dr-III | AAI08012.1 |
| *Felis catus* | PLA_2_ | Fc-III | XP_023097342.1 |
| *Homo sapiens* | PLA_2_ | Hsa-III | NP_056530.2 |
| *Ictidomys tridecemlineatus* | PLA_2_ | It-III | XP_005339912.1 |
| *Macaca nemestrina* | PLA_2_ | Mn-III | XP_011748880 |
| *Mesocricetus auratus* | PLA_2_ | Ma-III | XP_005068966.1 |
| *Microplitis demolitor* | PLA_2_ | Md-III | XP_008543925 |
| *Mus musculus* | PLA_2_ | Mm-III | NP_766379 |
| *Pan paniscus* | PLA_2_ | Pp-III | XP_003826977 |
| *Ovis aries* | PLA_2_ | Oa-IB | XP_004017463 |
| *Papio anubis* | PLA_2_ | Pa-III | XP_009215381 |
| *Rattus norvegicus* | PLA_2_ | Rn-III | NP_001099485 |
| *Spodoptera exigua* | PLA_2_ | Se-III | AZL90156 |
| *Homo sapiens* | PLA_2_ | Hsa-IV | NP_077734 |
| *Pan troglodytes* | PLA_2_ | Pt-IV | XP_001165694 |
| *Gorilla gorilla gorilla* | PLA_2_ | Ggg-IV | XP_004028114 |
| *Rattus norvegicus* | PLA_2_ | Rn-IV | NP_598235 |
| *Mus musculus* | PLA_2_ | Mm-IV | NP_032895 |
| *Homo sapiens* | PLA_2_ | Hsa-VI | NP_001004426 |
| *Gorilla gorilla gorilla* | PLA_2_ | Ggg-VI | XP_030861468 |
| *Pongo pygmaeus* | PLA_2_ | Poa-VI | XP_054325493 |
| *Macaca mulatta* | PLA_2_ | Mmu-VI | XP_028683552 |
| *Piliocolobus tephrosceles* | PLA_2_ | Pit-VI | XP_023077806 |
| *Rattus norvegicus* | PLA_2_ | Rn-XV | NP_001004277 |
| *Homo sapiens* | PLA_2_ | Hsa-VIII | NP_001242939 |
| *Pan paniscus* | PLA_2_ | Pp-VIII | XP_057158932 |
| *Gorilla gorilla gorilla* | PLA_2_ | Ggg-VIII | XP_030868607 |
| *Pan paniscus* | PLA_2_ | Pat-VIII | XP_057158930 |
| *Mus musculus* | PLA_2_ | Mm-XV | NP_598553 |
| *Homo sapiens* | PLA_2_ | Hsa-XV | 4X90_A |
| *Ochotona princeps* | PLA_2_ | Op-XV | XP_004584230 |
| *Frankliniella occidentalis* | PLA_2_ | FOPLA2-AsA | KAE8741843 |
| *Frankliniella occidentalis* | PLA_2_ | FOPLA2-BsB | XP_026293214 |
| *Frankliniella occidentalis* | PLA_2_ | FOPLA2-CiA | XP_026288906 |
| *Frankliniella occidentalis* | PLA_2_ | FOPLA2-DiB | XP_026285023 |
| *Frankliniella occidentalis* | Apolipophorin III | Fo-Apol | XP_026275826.1 |
| *Frankliniella occidentalis* | Defensin | Fo-Def | XP_026284438.1 |
| *Frankliniella occidentalis* | Lysozyme | Fo-Lyz | XP_026291031.1 |
| *Frankliniella occidentalis* | Transferrin 1 | Fo-Tra1 | XP_026287716.1 |
| *Frankliniella occidentalis* | dual oxidase | Fo-Duox | XP_026290106.1 |
| *Frankliniella occidentalis* | phenoloxidase-activating protease | Fo-PAP2A | XP_026283389.1 |
| *Frankliniella occidentalis* | phenoloxidase-activating protease | Fo-PAP2B | XP_026283307.1 |
| *Frankliniella occidentalis* | phenoloxidase-activating protease | Fo-PAP3 | XP_026272819.1 |
| *Frankliniella occidentalis* | phenoloxidase | Fo-PO1 | XP_026272742.1 |
| *Frankliniella occidentalis* | phenoloxidase | Fo-PO2A | XP_026273940.1 |
| *Frankliniella occidentalis* | phenoloxidase | Fo-PO2B | XP_026281547.1 |

**Table S2**. Primers used in this study

| **Genes** | **Orientation** | **Sequence (5ʹ - 3ʹ)** | **Uses** | **Anneal. Temp. (ºC)** | **Expected size (bp)** |
| --- | --- | --- | --- | --- | --- |
| *EF1* | Forward | TCAAGGAACTGCGTCGTGGAT | RT-qPCR | 52.0 | 130 |
|  | Reverse | ACAGGGGTGTAGCCGTTAGAG |  |  |  |
| *Fo-PLA_2_A* | Forward | ATTTGGGGGACAAGCAACCT | RT-qPCR | 54.0 | 168 |
|  | Reverse | CACCCGCTCACGATTCACTA |  |  |  |
| *Fo-PLA_2_B* | Forward | ACTATCCCCTTGGTTGGGAA | RT-qPCR | 52.0 | 275 |
|  | Reverse | CAGTGTAAGCACCCTTCCAG |  |  |  |
| *Fo-PLA_2_C* | Forward | CTCCCGTTCAGAAGACGATG | RT-qPCR | 52.0 | 364 |
|  | Reverse | TCGTTTTCTGTCGCAGTTGA |  |  |  |
| *Fo-PLA_2_D* | Forward | GGTGGCTCTGTTAAAGCCCT | RT-qPCR | 52.0 | 324 |
|  | Reverse | TGAGGGAGTAAGGCTCCACA |  |  |  |
| *T7+ Fo-T7PLA2A* | Forward | TAATACGACTCACTATAGGGAGAATTTGGGGGACAAGCAACCT | RNAi | 54.0 | 168 |
|  | Reverse | TAATACGACTCACTATAGGGAGACACCCGCTCACGATTCACTA |  |  |  |
| *T7+ Fo-T7PLA2B* | Forward | TAATACGACTCACTATAGGGAGAACTATCCCCTTGGTTGGGAA | RNAi | 54.0 | 275 |
|  | Reverse | TAATACGACTCACTATAGGGAGACAGTGTAAGCACCCTTCCAG |  |  |  |
| *T7+ Fo-T7PLA2C* | Forward | TAATACGACTCACTATAGGGAGACTCCCGTTCAGAAGACGATG | RNAi | 54.0 | 364 |
|  | Reverse | TAATACGACTCACTATAGGGAGATCGTTTTCTGTCGCAGTTGA |  |  |  |
| *T7+ Fo-PLA2D* | Forward | TAATACGACTCACTATAGGGAGAGGTGGCTCTGTTAAAGCCCT | RNAi | 54.0 | 324 |
|  | Reverse | TAATACGACTCACTATAGGGAGATGAGGGAGTAAGGCTCCACA |  |  |  |
| *Duox* | Forward | CAGCAGGTCACCGAACTCAT | RT-qPCR | 52.0 | 193 |
|  | Reverse | AGCTCGTCATTCTCGCTACG |  |  |  |
| *PAP2A* | Forward | ACGGCGAGGGAATTATCGAC | RT-qPCR | 52.0 | 311 |
|  | Reverse | CAACACGTTGCTCTGGTGAC |  |  |  |
| *PAP2B* | Forward | CAACGACAAGAGCCTCCACA | RT-qPCR | 52.0 | 248 |
|  | Reverse | GGCGAGTCTTGCGGAGTAAT |  |  |  |
| *PAP3* | Forward | GGATCTCTCTGCTCGCCATC | RT-qPCR | 52.0 | 133 |
|  | Reverse | GACTCGCGCTACCTGTTCG |  |  |  |
| *PO1* | Forward | TCAACCCCTACCTGTTCGTG | RT-qPCR | 52.0 | 188 |
|  | Reverse | GATGATGATGGGCAGTCGCT |  |  |  |
| *PO2A* | Forward | GTACCTGTTCGACAGACCCG | RT-qPCR | 52.0 | 287 |
|  | Reverse | CGAAGAAATGCCAGAAGCGG |  |  |  |
| *PO2B* | Forward | TTCTTCGAAGCGGCAAACAC | RT-qPCR | 52.0 | 162 |
|  | Reverse | GAAGTACTGGGTCTGCTCGG |  |  |  |
| *Apol* | Forward | ACCGGGCCCCAGGCCTCCCG | RT-qPCR | 52.0 | 133 |
|  | Reverse | CCCTGGCGTCGGCGTCGTCC |  |  |  |
| *Def* | Forward | ATGCAGAGCCTCACCTTAGG | RT-qPCR | 54.0 | 153 |
|  | Reverse | CCGGGGCGAGCGGCCAGTGC |  |  |  |
| *Lyz* | Forward | TATGGGAGCGGTGTACTTTA | RT-qPCR | 52.0 | 139 |
|  | Reverse | CCATCGGTGTTTACCGTGTT |  |  |  |
| *Tra1* | Forward | GACTTCGGCGTCCTCACCGCG | RT-qPCR | 52.0 | 171 |
|  | Reverse | GCCGCTGAGGAGCCCGGTGT |  |  |  |

**Table S3**. Probe sequences used in FISH assay

| **Genes** | **Probes** | **Sequence (5ʹ - 3ʹ)** |
| --- | --- | --- |
| *Fo-PLA_2_A* | Antisense | Marina blue-TTT-CACCCGCTCACGATTCACTA |
|  | Sense | Marina blue-AAA-TAGTGAATCGTGAGCGGGTG |
| *Fo-PLA_2_B* | Antisense | Rodamine6G-TTT-CAGTGTAAGCACCCTTCCAG |
|  | Sense | Rodamine6G-AAA-CTGGAAGGGTGCTTACACTG |
| *Fo-PLA_2_C* | Antisense | FITC-TTT-TCGTTTTCTGTCGCAGTTGA |
|  | Sense | FITC-AAA-TCAACTGCGACAGAAAACGA |
| *Fo-PLA_2_D* | Antisense | FITC-TTT-TGAGGGAGTAAGGCTCCACA |
|  | Sense | FITC-AAA-TGTGGAGCCTTACTCCCTCA |
